# Primary healthcare and school health service utilisation by adolescents and young adults in KwaZulu-Natal, South Africa

**DOI:** 10.1186/s12913-019-4559-2

**Published:** 2019-11-28

**Authors:** Aoife M. Doyle, Lerato Mchunu, Olivier Koole, Sandile Mthembu, Siphephelo Dlamini, Nothando Ngwenya, Jane Ferguson, Janet Seeley

**Affiliations:** 1 Department of Infectious Disease Epidemiology, London School of Hygiene and Tropical Medicine, Keppel Street, London, WC1E 7HT UK; 2 Africa Health Research Institute, Durban, KwaZulu-Natal South Africa

**Keywords:** South Africa, Adolescent, Health services, Young adult

## Abstract

**Background:**

Young people aged 10–24 years are a vulnerable group with poor health service access relative to other populations. Recent South African initiatives, the She Conquers campaign, the Integrated School Health Policy and the Adolescent & Youth Health Policy, include a focus on improving the breadth and quality of youth-friendly health service delivery. However, in some settings the provision and impact of scaled-up youth friendly health services has been limited indicating a gap between policy and implementation. In this study we reviewed existing sources of data on health service utilisation to answer the following question: ‘What health conditions do young people present with and what services do they receive at public health clinics, mobile clinics and school health services?’

**Methods:**

We conducted a retrospective register review in three purposively selected primary healthcare clinics (PHCC), one mobile clinic, and one school health team in Hlabisa and Mtubatuba sub-districts of uMkhanyakude District, KwaZulu-Natal, South Africa. The focus was service utilisation for any reason by 10–24 year olds. We also conducted descriptive analysis of pre-existing data on service utilisation by young people available from the District Health Information System for all 17 PHCC in the study sub-districts.

**Results:**

Three quarters of 4121 recorded young person visits in the register review were by females, and 40% of all young person visits were by females aged 20–24 years. The most common presenting conditions were HIV-related, antenatal care, family planning, general non-specific complaints and respiratory problems (excluding TB). There were relatively few recorded consultations for other common conditions affecting young people such as mental health and nutritional problems. Antibiotics, antiretrovirals, contraceptives, vitamins/supplements, and analgesics were most commonly provided. Routine health registers recorded limited information, were often incomplete and/or inconsistent, and age was not routinely recorded.

**Conclusions:**

Measuring morbidity and service provision are fundamental to informing policy and promoting responsive health systems. Efforts should be intensified to improve the quality and completeness of health registers, with attention to the documentation of important, and currently poorly documented, young people’s health issues such as mental health and nutrition.

## Background

Young People (YP) aged 10–24 years have relatively poor access to health services. Ensuring that YP can access high-quality preventive and curative health services is important for their current and future health outcomes, and for the health outcomes of their children [1, 2]. The provision of appropriate services for YP requires an understanding of the important health concerns in a community. Yet often the local context of YP’s health and well-being is poorly understood. Up until recently, the focus of health services for YP in sub-Saharan Africa has been on sexual and reproductive health services including HIV testing, care and treatment. These remain important, but mental health, trauma-care related to violence/accidents, advice and early intervention for alcohol and substance use, and nutrition are important health issues for YP and have historically largely been ignored [2].

These concerns are addressed in recent South African (SA) initiatives, the Integrated School Health Policy [3] and the Adolescent & Youth Health Policy [4], which include a focus on health service delivery. However, in some settings the provision and impact of scaled-up youth friendly health services has been limited [5], and there is often a gap between policy and adequate youth-friendly service provision [2, 6]. There remains a paucity of evidence on the effectiveness of delivering health services outside of the primary health care clinic (PHCC) [7–10], and limited evidence on the most appropriate and cost-effective services to offer YP in a low-resource setting.

The ability to measure YP morbidity, and health service quality and provision is fundamental to inform policy. In SA, the district health information system (DHIS) is designed to collect and collate data on key health outcomes and the utilisation of essential services. Collated data are then available for use by both the PHCC and district management teams. However, a 2008 mixed methods study of DHIS and its use in 10 rural districts in KwaZulu-Natal revealed that the culture of information use was weak within the PHCC with data collection and collation perceived to be a high work burden [11]. The Ideal Clinic programme is a 2013 Department of Health initiative that aims to transform PHCC to meet a defined set of national standards [12]. These standards include targets related to data management such as ‘Relevant DHIS registers are available and are kept up to date’, and others focusing on YP such as ‘Facility has a brief profile of adolescents and youth in its catchment area including their challenges’ [12].

In this study, we sought to answer the question ‘What health conditions do YP present with and what services do they receive at public health clinics, mobile clinics and school health services?’

## Methods

In 2017, the mixed methods ‘Health Services for Young People’ study was conducted in uMkhanyakude, one of the poorest districts in KwaZulu-Natal [13]. The district has high unemployment and low literacy rates, limited access to piped water and electricity, and poor road infrastructure [14]. In 2012, the provincial HIV prevalence in KwaZulu-Natal was estimated to be the highest in the country at 16.9% [15]. PHCC provide a comprehensive package of health care services, however, a focus on HIV services in more recent years has meant that other essential primary health care services such as non-communicable diseases, maternal health, nutrition and health promotion have been less well implemented. The provision of mental health, oral and eye health and rehabilitation is especially limited [16].

We report here on the quantitative component of the study. The qualitative component of the study has been reported elsewhere [17]. Quantitative data collection and analysis was made-up of two parts:

### Retrospective register review

We reviewed paper registers in three purposively selected PHCC, one mobile clinic, and one school health team in Hlabisa and Mtubatuba sub-districts of uMkhanyakude to assess clinic attendance for any reason by males and females aged 10–24 years.

During the register review, trained study nurses extracted relevant data from each clinic’s paper registers and entered the data into pre-programmed REDCap forms on tablets. Study nurses were encouraged to record the completeness and consistency of the data within each register.

Hlabisa and Mtubatuba sub-districts have a total of 17 PHCC: three urban, three semi-urban and eleven rural. The study clinics were selected from those with whom the Africa Health Research Institute (AHRI) Population Intervention Programme (PIP) already had established regular collaboration [18, 19]. Clinics were purposively selected to be broadly representative of the medium and small semi-urban and rural clinics in the study area. Clinic 1 was a medium semi-urban clinic; clinic 2 was a medium rural clinic; and clinic 3 was a small rural clinic.

One of the two mobile clinic teams operational in Mtubatuba sub-district was purposively selected to participate in the register review, as was one of the two sub-district school health teams.

In each study clinic, two study nurses aimed to review the following main registers (Table 1):

**Table 1:** Registers and data sources reviewed

Data source (time period)	Location	Unit recorded on register	Data elements relevant for this study	Type of field	Expected coverage
Daily clinic ‘pink’ registers (May 15, July 15, Sep 15, Nov 15, Jan 16 , Mar 16)	PHCC and mobile clinics	Client consultation			All clinic consultations
			Age		
			Gender		
			Provisional diagnosis	Free text	
			Treatment/management	Free text	
PHCC comprehensive tick register (May-Sept 16)	PHCC	Client consultation			All clinic consultations
			Family planning acceptor <18 years	Tick box	
			Diabetes client <18 years new	Tick box	
			Mental health client < 18 years	Tick box	
			Td dose at 12 years	Tick box	
			ANC 1st visit <18 years	Tick box	
			Other reasons for consultation/service provided/clinical outcome - ~50 categories	Tick boxes	
Integrated School Health Programme Daily Register (Jul 15, Sep 15, Nov 15, Jan 16, Mar 16, May 16)	School health team (SHT)	Client consultation			All consultations with SHT
			Gender of student	Tick box	
			Screening in grade 8	Tick box	
			Screening in grade 10	Tick box	
			12 year tetanus vaccination given	Tick box	
			Other reasons for consultation- 13 categories	Tick boxes	
			Treatment given/referral to services- 28 categories including ‘Td given’	Tick boxes	
District Health Information System (May-Sep 16)	District	Consultations per clinic per month			All clinic consultations
			Family planning acceptor <18 years	Number	
			Diabetes client <18 years new	Number	
			Mental health client < 18 years	Number	
			Td dose at 12 years	Number	
			ANC 1st visit <18 years	Number	

#### Daily clinic ‘pink’ registers

Registers used by clinic staff to record the age, gender of the clients, the presenting condition, and the treatment given. The daily clinic register review took place between January and March 2017 with registers reviewed for 6 months of a one-year period (May 2015, July 2015, September 2015, November 2015, January 2016, March 2016). This was the most recent period prior to the April 2016 replacement of the daily clinic registers with the PHCC comprehensive tick register. Daily clinic register review in clinic 1 was not possible as the daily clinic registers had been moved to the district hospital (Hlabisa) for filing. Following piloting, the study nurses reviewed the conditions being recorded and grouped them into 38 categories. Similarly, 33 categories were created for treatment and services provided (Additional file 1: **Table S1**).

#### PHCC comprehensive tick registers-

Since April 2016, these registers have been used to record attendances for select conditions and treatments. Tick register data were extracted over 4 days between February and April 2017 for the months May–September 2016. Age was not recorded on these registers so to estimate the attendance of YP, study nurses extracted data where one of the following was ticked: Family planning acceptor < 18 years, Diabetes client < 18 years new, Mental health client < 18 years, Td dose at 12 years, ANC 1st visit < 18 yrs.

Other disease or service specific registers that were present in the clinics such as TB, ART and delivery registers were not consulted.

#### Integrated school health Programme daily register

The school health team (SHT) comprises two nurses who provide periodic services at schools. They conduct screening of learners in grade 8 (approximately aged 14 years) and grade 10 (approximately aged 16 years) and provide advice, treatment (primarily analgesics) and referrals for screened students and for other learners who present to the nurses. The SHT had their own register to record the occurrence of health screenings and immunizations. Multiple conditions and/or treatments could be ticked. The school health register recorded the gender of the learner, the reason for seeing the nurse and the treatment given. Age was not recorded. Study nurses extracted data where the register indicated that the learner received one of the following services delivered to learners in our target age range: grade 8 screening, grade 10 screening and tetanus vaccination at age 12. The school health registers were reviewed for: July 2015, September 2015, November 2015, January 2016, March 2016, May 2016. During these months the school health team provided services in 7 schools.

### Other sources of data on YP health service utilisation

In order to get a better understanding of whether the patterns of health service utilisation observed in our three study clinics were broadly representative of utilisation patterns observed across the district, we conducted secondary analysis of data from one additional source **(**Table 1**)**.

We analysed **District Health Information System (DHIS)** adolescent health indicators for the 17 PHCC in Mtubatuba and Hlabisa sub-districts for the period May 2016–September 2016. The DHIS database did not contain detailed information on treatment and services provided according to age and gender. PHCC-level DHIS data was a summary of the comprehensive tick register data that PHCC submitted monthly to the district. At each PHCC a weekly tally sheet was available for collating daily/weekly totals and a monthly summary sheet was used for submission to the sub-district/district [20].

### Analysis

We analysed routine health facility and school health data to describe the health conditions that young people presented with and the services that they received. The data were described according to age group, gender, and clinic. In bivariate analysis, chi-squared tests were used to compare the observed proportions against the null hypothesis of equal distribution of outcomes across categories e.g. equal number of clinic attendances by males and females. Comprehensive tick register data and DHIS data were compared to assess internal consistency. For each of the target months, we reviewed the comprehensive tick registers and manually counted up the number of ticks each month each of the target adolescent health indicators. We then compared this tick register monthly total to the monthly total as recorded in the DHIS.

## Results

### Number of YP attending fixed and mobile clinics (daily clinic register)

Over 6 months in each of three clinics (18 months in total), there were 4121 **daily clinic register** records of clinic attendance by YP **(**Table 2**)**. Mobile clinic 1 visited 20 communities during the time periods of interest with 2 to 148 YP clients recorded in each community.

**Table 2:** YP client visits by gender and age group. Source: Daily clinic registers (6 months, 2015/16)^a^

		Clinic 2	Clinic 3	Mobile clinic
Median (IQR) YP visits/facility/month		311 (201, 509)	299 (255, 311)	114 (112, 122)
Total		1846	1591	684
Age group	10–14 yrs	21.1%	16.1%	15.2%
	15–19 yrs	34.2%	37.3%	29.2%
	20–24 yrs	44.7%	46.6%	55.6%
Gender	Female	73.8%	75.9%	88.2%
	Male	26.2%	24.1%	11.8%
Female	10–14 yrs	10.6%	8.3%	9.2%
	15–19 yrs	26.4%	29.4%	26.2%
	20–24 yrs	36.8%	38.2%	52.8%
Male	10–14 yrs	10.6%	7.8%	6.0%
	15–19 yrs	7.8%	7.9%	3.1%
	20–24 yrs	7.9%	8.5%	2.8%

^a^*Registers were reviewed for the months May 2015, July 2015, September 2015, November 2015, January 2016, and March 2016. March 2016 was excluded when calculating the mean, median, interquartile range (IQR) number of clients per month for all clinics as the study nurses were not able to find all the registers for this month and there were only four recorded YP attendances in the available registers. Manual register review took place January–March 2017*

### Age and gender distribution of attendees (daily clinic register)

Three quarters of YP clinic visits were by females and 40% by females aged 20–24 years. A higher proportion of client visits were by females compared to males in the age groups 15–19 years and 20–24 years (*p* < 0.001). This gender imbalance in client attendances was not seen in the age group 10–14 years where approximately half of YP client visitors were male (*p* = 0.44). Given the overall gender distribution of clients, the mobile clinic had a lower than expected proportion of male clients (Table 2, *p* < 0.001).

### Diagnosis and presenting conditions (daily clinic register)

**Fig. 1:**
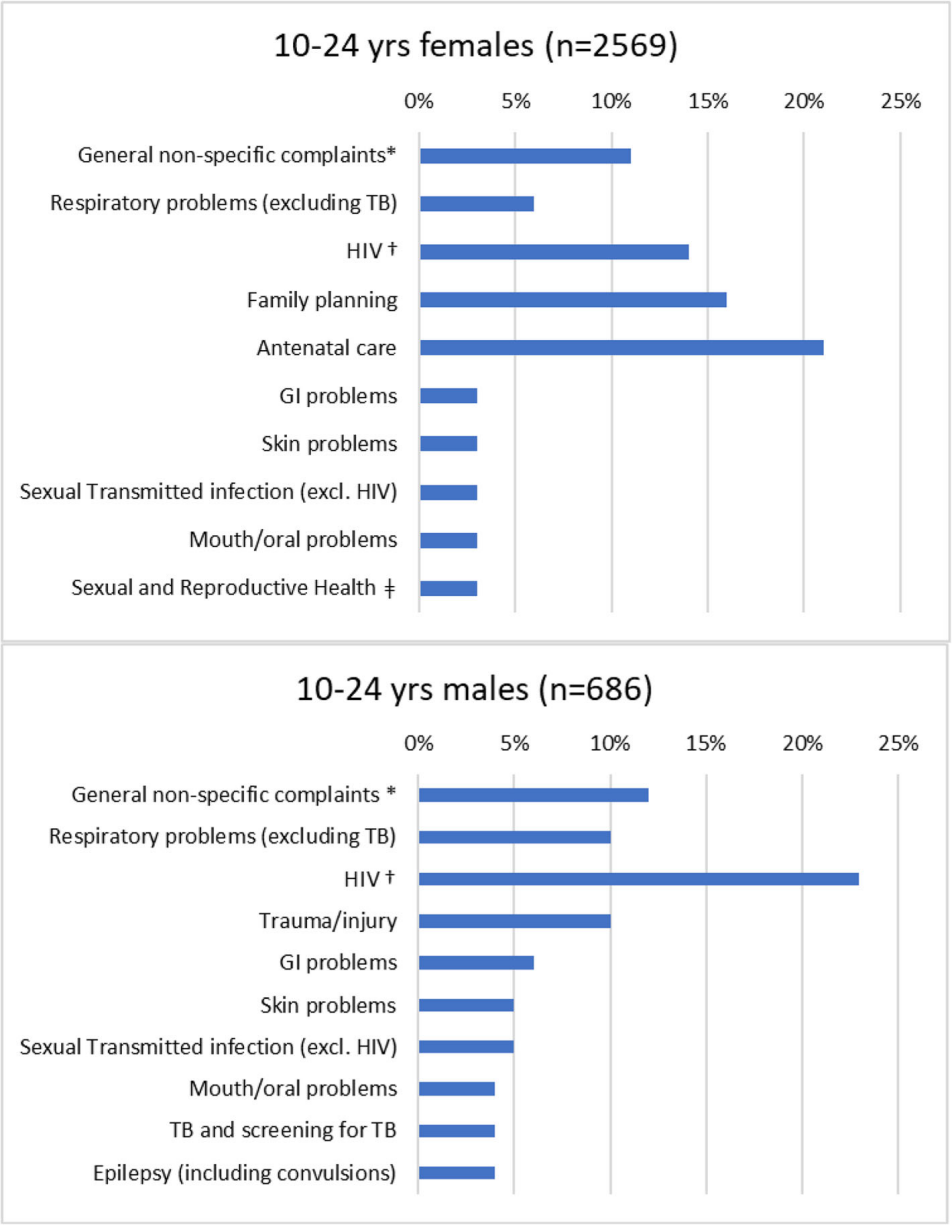
Top 10 provisional diagnosis/presenting conditions by gender. (Daily clinic registers, 2 PHCC (6 months, 2015/16)). *Including abdominal pains/cramps, headache, backache, general body pains, painful legs, dizziness, fatigue, poor appetite, heart palpitations, nausea/vomiting, general body malaise, common cold/flu. †Including testing, diagnosis, treatment, follow-up, treatment failure. ǂ PAP, dysmenorrhoea, spotting, PV bleeding, pregnancy testing

The most common diagnosis or presenting conditions on the **daily clinic registers** at the two fixed PHC (clinics 2 & 3) were HIV-related conditions, antenatal care, family planning, general non-specific complaints (e.g. headache, body aches, nausea, common cold etc.) and respiratory problems (excluding TB) **(**Fig. 1**,** Table 3). There were relatively few recorded consultations for other conditions affecting YP such as mental health issues (8 consultations) and nutritional problems (1 consultation). There were 4 abortions recorded, and 33 deliveries (11 among 15–19 year olds and 22 among 20–24-year-old females).

**Table 3:** Top 5 provisional diagnoses/presenting condition and top 5 treatments according to age group and gender (Pink register data, 2 PHC, 6 months in 2015/16)

	Total				Females				Males			
	10-14 yrs	15-19 yrs	20-24 yrs	Total	10-14 yrs	15-19 yrs	20-24 yrs	Total	10-14 yrs	15-19 yrs	20-24 yrs	Total
	(*n*=646)	(*n*=1224)	(*n*=1567)	(*n*=3437)	(*n*=327)	(*n*=956 )	(*n*=1286)	(*n*=2569 )	(*n*=319 )	(*n*=268)	(*n*=281)	(*n*=868)
Provisional diagnoses/presenting condition												
General non-specific complaints	**11.6%**	**11.0%**	**10.3%**	**10.8%**	**14.1%**	**10.7%**	**9.6%**	**10.6%**	**9.1%**	**12.3%**	**13.5%**	**11.5%**
Skin problems	**7.7%**	3.4%	2.0%	3.6%	**8.0%**	2.6%	2.2%	3.1%	**7.5%**	6.0%	1.4%	**5.1%**
Respiratory problems (excluding TB)	**13.5%**	**7.3%**	4.9%	**7.4%**	**16.5%**	6.2%	3.9%	**6.3%**	**10.3%**	**11.2%**	**9.6%**	**10.4%**
TB and screening for TB	2.0%	1.6%	2.5%	2.1%	2.5%	0.8%	1.6%	1.4%	1.6%	4.5%	6.8%	4.2%
HIV^a^	**28.2%**	**10.2%**	**15.3%**	**15.9%**	**22.0%**	**9.0%**	**15.1%**	**13.7%**	**34.5%**	**14.6%**	**16.4%**	**22.5%**
GI problems	4.3%	4.4%	3.2%	3.8%	4.3%	3.7%	2.4%	3.1%	4.4%	**7.1%**	6.8%	6.0%
Sexual Transmitted infection (excl. HIV)	0.5%	3.2%	**5.2%**	3.6%	0.9%	3.0%	3.6%	3.0%	0.0%	3.7%	**12.5%**	5.2%
Sexual and Reproductive Health^b^	0.8%	6.1%	4.7%	4.5%	1.5%	**7.7%**	**5.7%**	2.9%	0.0%	0.4%	0.4%	0.2%
Family planning	0.2%	**9.8%**	**18.0%**	**11.7%**	0.3%	**12.1%**	**21.8%**	**15.5%**	0.0%	1.5%	0.7%	0.7%
Antenatal care	2.5%	**21.7%**	**16.2%**	**15.5%**	4.6%	**27.6%**	**19.6%**	**20.7%**	0.3%	0.4%	0.4%	0.4%
Trauma/injury	**7.6%**	4.8%	2.1%	4.1%	**5.8%**	2.5%	0.9%	2.1%	**9.4%**	**13.1%**	**7.8%**	**10.0%**
Treatments												
Antibiotics	**23.5%**	**20.6%**	**18.8%**	**20.3%**	**25.4%**	**18.0%**	**15.3%**	**17.6%**	**21.6%**	**29.9%**	**34.5%**	**28.3%**
Anti-inflammatory	0.3%	1.4%	1.7%	1.3%	0.6%	1.3%	1.1%	1.1%	0.0%	1.9%	**4.3%**	2.0%
Panadol/Aspirin	**10.5%**	**8.7%**	**7.2%**	**8.4%**	**12.5%**	**8.4%**	**6.8%**	**8.1%**	**8.5%**	**9.7%**	**9.3%**	**9.1%**
Anti-retroviral treatment (ART)	**28.3%**	**10.2%**	**14.6%**	**15.6%**	**21.7%**	**9.2%**	**14.3%**	**13.4%**	**35.1%**	**13.8%**	**16.0%**	**22.4%**
TB treatment	0.5%	0.5%	1.9%	1.1%	0.9%	0.1%	1.2%	0.7%	0.0%	1.9%	**5.3%**	2.3%
Epileptic treatment	2.8%	0.0%	1.4%	1.9%	2.8%	1.4%	0.8%	1.3%	2.8%	4.5%	**4.3%**	3.8%
Vitamins and supplements	3.4%	**18.3%**	**15.4%**	**14.2%**	**5.8%**	**22.9%**	**18.4%**	**18.5%**	0.9%	1.9%	1.8%	1.5%
Vaccination	**5.0%**	3.1%	1.0%	2.5%	4.9%	2.3%	0.5%	1.7%	**5.0%**	**6.0%**	3.2%	**4.7%**
Minor procedures/dressing	4.5%	2.7%	2.7%	3.0%	2.5%	2.5%	3.0%	2.7%	**6.6%**	3.4%	1.4%	3.9%
Symptomatic treatment^c^	**5.9%**	4.9%	3.4%	4.4%	**6.7%**	3.8%	3.7%	4.1%	**5.0%**	**8.9%**	1.8%	**5.2%**
Family planning	0.2%	**11.0%**	**18.9%**	**12.5%**	0.3%	**13.6%**	**22.9%**	**16.5%**	0.0%	1.5%	0.7%	0.7%

^a^Including testing, diagnosis, treatment, follow-up, treatment failure

^b^PAP, dysmenorrhoea, spotting, PV bleeding, pregnancy testing

^c^loperamide, cough mixture as DPH or Cocillona, mouth wash, calamine lotion, castor oil, lactulose, anusol, activated charcoal, antiseptic, benzoic acid

**Fig. 2:**
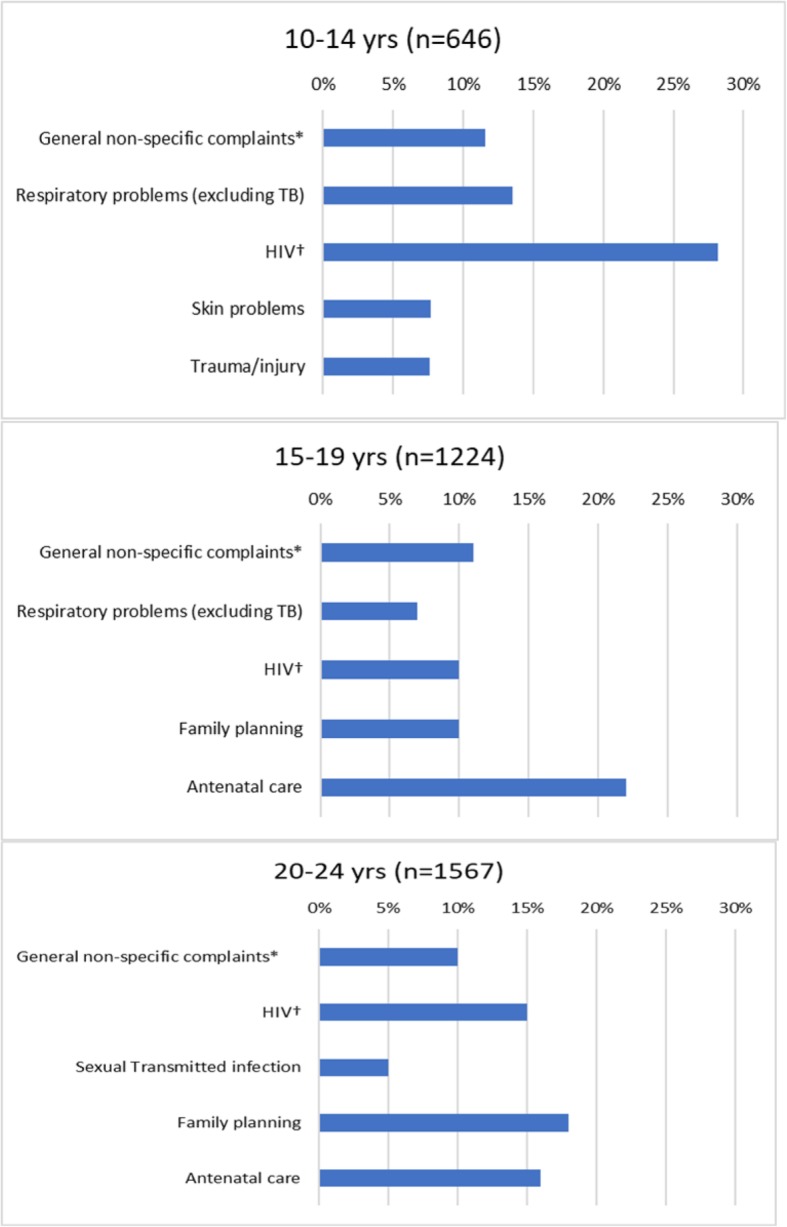
Top 5 provisional diagnosis/presenting conditions by age group. (Daily clinic registers, 2 PHCC (6 months, 2015/16)). *Including abdominal pains/cramps, headache, backache, general body pains, painful legs, dizziness, fatigue, poor appetite, heart palpitations, nausea/vomiting, general body malaise, common cold/flu. †Including testing, diagnosis, treatment, follow-up, treatment failure

Females presented most frequently for antenatal care (20.7% of all female attendances) and, males for HIV (22.5% of all male attendances) **(**Fig. 1**)**. The most frequent presenting condition for 10–14 year olds was HIV, and for those aged 15 or older was antenatal care and family planning (Fig. 2). At the mobile clinic, the main presenting conditions were family planning (45.0%), general non-specific complaints (15.4%) and gastrointestinal problems (8.6%) (data not shown).

### Treatment and services (daily clinic register)

The most common treatments and services recorded on the **daily clinic registers** in clinics 2 and 3 were antibiotics, antiretroviral drugs (ARVs), vitamins and supplements, family planning, and Panadol/aspirin. Family planning was the third most common service provided for females and the most common treatment/service for females aged 20–24 years. Antibiotics was the most common treatment for 10–14-year-old females and overall for males (Table 3**,** Additional file 2: **Table S2**).

In the mobile clinic, the most common treatments and services were family planning, antibiotics, Panadol/aspirin, and symptomatic treatment. The distribution of treatment varied by clinic, with ARVs accounting for a higher proportion of treatments being provided at visits in clinic 2 compared to clinic 3 (21.3% vs. 9.0%, *p* < 0.001). The provision of family planning accounted for 12.9% of female treatments in clinic 2, 20.7% in clinic 3 and 51.1% in mobile clinic 1.

### District level information on adolescent health (PHC comprehensive tick registers and DHIS data)

**Fig. 3:**
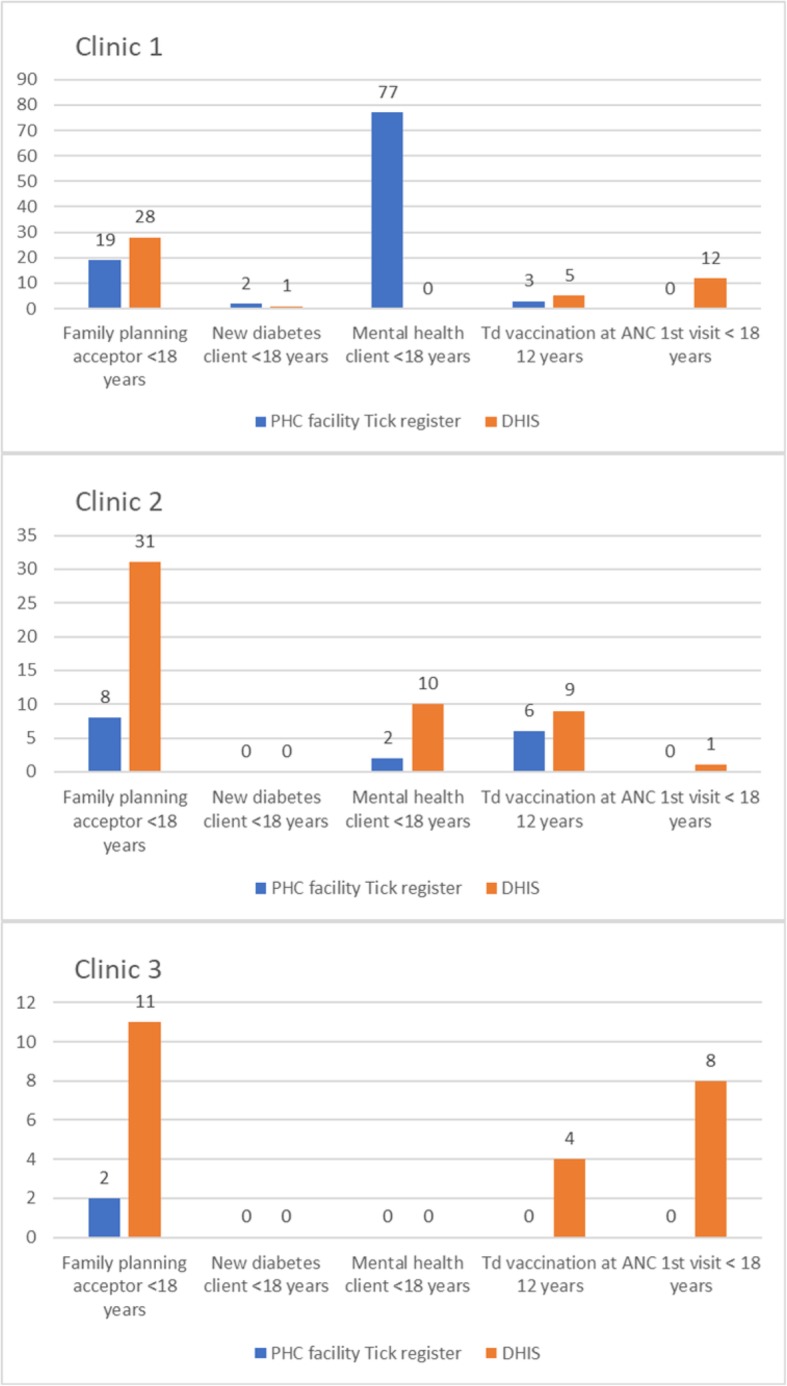
Adolescent health indicators: PHCC comprehensive tick registers vs. DHIS data (three PHCC, May-Sept 2016)

In the PHCC comprehensive tick registers, a total of 115 clients were seen for adolescent conditions and services (see methods) over a 5-month period **(**Fig. 3**).** In clinic 1, most clients (69/97) visited during August, and the majority were recorded as mental health clients < 18 years (77/97). The DHIS data for the same 5 months in the same clinics showed a similar pattern **(**Fig. 3**)** except for the notably lower number of mental health clients < 18 years in clinic 1.

### School health registers

In 6 months of school health registers, a total of 209 learners in seven schools were recorded as having received one or more of the services of interest: Grade 8 screening (77 learners), Grade 10 screening (78 learners), and tetanus vaccination at age 12 years (100 learners) (Additional file 3: **Table S3**). A higher proportion of learners receiving services were females (70.3%) compared to males (29.7%) (*p* < 0.001). The SHT completed the registers in each school over a period of 1–3 days and the number of learners recorded in each school ranged from 1 to 97.

### Limitations of the registers, and data quality and completeness

Some of the registers were missing or had been sent to the District office for filing and were not available. Study nurses noted that the data in the tick registers were often inconsistent. For example, where ‘family planning acceptor <18 years’ was ticked, the method of contraception provided was not always ticked. In the school health registers, there were inconsistencies e.g. there were two places in the register where the nurse should indicate that the tetanus vaccination had been given and they were both ticked for only 68/100 learners. In some instances, the daily clinic registers recorded a much higher number of YP client visits for a particular reason when compared to the tick registers. The time periods reviewed for the two registers were different, but this suggests that there may be considerable underreporting of, for example, adolescent family planning clients in the tick register. Furthermore, there were no instances of ANC first visit <18 years in the tick registers that we reviewed. The comparison of tick register data from the clinics and DHIS data at the District for the same months and same clinics revealed many discrepancies **(**Fig. 3**).** Possible explanations include missing registers, transcription error by study nurses, recording errors by PHC nurses, and updating/correcting of tick register data by PHC staff prior to submission to the district. Even if the registers were complete and accurate they would still present some issues for the measurement of health service utilisation, and hence estimation of YP morbidity, as neither age nor gender are recorded in the comprehensive tick register and age is not recorded on the school health register.

## Discussion

Primary healthcare consultations for 10–24 year olds in rural KwaZulu-Natal were primarily for HIV, family planning, antenatal care, and general non-specificcomplaints. A direct comparison with sources of morbidity data is not possible as different age-groups, and categories of morbidity are used. However, the health conditions that YP presented with in our study were consistent with known patterns of morbidity. For example, in African low and middle-income countries the top five causes of loss of adolescent disability-adjusted life years (DALYs) were lower respiratory infections, diarrheal diseases, meningitis, iron-deficiency anaemia and HIV/AIDS [21]. In the 2013 South African General Household Survey, the most frequently self-reported illnesses by youth (15–34 years) in the month before the survey were flu/ARTI, diarrhoea, epilepsy, TB and high blood pressure [22]. In 2010, a survey of consultations (age 0–79 years) in primary care in four provinces in the north and west of South Africa, found that primary care was dominated by non-communicable chronic diseases and that HIV/AIDS and TB were common. Consultations by YP had similar patterns with, for example, with a high proportion of visits for women’s health issues (family planning) [23].

Given the high prevalence of mental health disorders in YP, the low number of diagnoses and/or treatments of these conditions is notable. Other studies in SA have also observed low levels of mental health consultations in primary health care settings [23]. Mental health disorders, interpersonal violence including rape, and abortion are often stigmatised which may deter health service attendance. Other health conditions that are known to be important for adolescents such as nutrition, violence, and abortion did not emerge as common reasons for PHCC consultation in this study and suggest that either YP do not present for these conditions as often as they should and/or the diagnosis or recording of these conditions is sub-optimal.

Consultation patterns varied by gender and age. As we do not know the number of YP in the population who needed services, we cannot determine whether males were attending less frequently than females although there is evidence from studies in South Africa that younger men are less likely to seek care when ill, and 15–19 year olds are less likely to seek care than 20–24 year olds [24].

Our review of the school health registers suggests that a wide range of services are being offered to YP, including onward referral to health and social services, but the extent and coverage of services appears limited. It is not clear whether it is the services provided or the registration of those services that is limited. The KwaZulu-Natal provincial Ideal Clinic Realisation and Maintenance (ICRM) plan includes targets for the expansion of the school health programme with the prioritization of the most deprived wards [14]. The higher proportion of females compared to males on the school health registers was unexpected and a more detailed review of the implementation of school health services in uMkhanyakude is warranted.

This review of primary healthcare registers highlights challenges associated with both the appropriateness of data collected on YP and the accuracy and completeness of records. The pre-April 2016 daily clinic registers provided the most useful information, allowing us to summarise both presenting condition, and treatment and service, according to age and gender. The new comprehensive tick register (April 2016-present) no longer records the age and gender of the client and, to reduce the recording burden on clinic staff, records only when a YP presents for a number of key health conditions. The use of ‘less than 18 years’ as an age category means that it is impossible to determine if the client was aged 10–18 years or less than 10 years of age.

In 2017, the SA Department of Health revised the National Indicator Data Set and several new adolescent-specific indicators have been recommended for collection at PHC and hospital settings (Table 4, (25). These indicators prioritise SRH and mental health, two of the most important causes of morbidity and mortality among YP [21].

**Table 4:** South African adolescent health data elements (DE) and indicators (I) recommended for use from 1st April 2017

PHC headcount 10–19 y (DE)	
PHC utilisation rate 10–19 y (I)	
Mental health clients under 18 years (DE)	
Mental health separations under 18 years (DE)^a^	
Male medical circumcision 10–14 years (DE)	
Delivery 10–14 years in facility (DE)^b^	
Delivery 15–19 years in facility (DE)^b^	
Delivery in 10 to 19 years in facility rate (I)^b^	
Termination of pregnancy 10–19 years (DE)	

^a^Client under 18 years admitted for mental health conditions [25].

^b^Delivery where the mother is 10–14 years or 15–19 years old. These deliveries are done in facilities under the supervision of trained medical/ nursing staff [25].

While the updating of these indicators is a positive step, even if these data are accurately and consistently fed back at district level, they will provide only a very crude and limited summary of adolescent health in the district. More comprehensive monitoring of YP’s health which considers the epidemiology of disease in that age group is required. For example, obesity is becoming an important health issue in middle-income countries such as South Africa [26] yet, this is not captured in any of the registers reviewed. School learner overweight and underweight rates are included as school health indicators [25] but the calculation of these rates would not have been possible using the school health registers reviewed. Substance use, and violence, which were highlighted as major issues for YP during accompanying qualitative work [27] may be monitored more appropriately outside of the PHCC.

One limitation of our study is that we did not have denominator data i.e. the number of YP according to gender and age who needed services, and health care utilisation may not be related to the need which is measured as morbidity or health status. We did not collect information on clients of other ages, so we were not able to comment on morbidity and service utilization of YP compared to other population groups. Our register review did not include disease specific registers e.g. HIV and TB registers, and it is possible that those registers are of a higher quality. Our review covered only six out of 12 months in a calendar year and we may have missed important temporal differences. The study PHCC were purposively selected and might not be representative of all PHCC in uMkhanyakude. However, we did review DHIS data for a wider range of clinics and the findings were similar. The study clinician and nurses created categories to group the conditions mentioned in the registers. There may have been some inappropriate groupings and/or inconsistent coding of the register data by the study nurses. If some errors in coding did occur, then we expect that this would have had a minimal effect on the service utilisation findings.

## Conclusion

To our knowledge this is the first publication which has looked specifically at the recording of YP health and services in primary healthcare registers in South Africa. Our study has limited external validity beyond the two districts where registers were reviewed, however, our observations on the limitations of the youth indicators will be relevant nationally. The service utilization data described here and our observations on the implementation of the health registers will help to guide future policy in this area. For example, we suggest that primary health care services for common non-SRH conditions that affect YP (e.g. mental health and nutrition) should be enhanced, and greater numbers of young men encouraged to access services. Recent changes in the health registers, which aim to decrease the recording burden on healthcare providers, mean that very limited data on health service utilisation among YP is available to inform service provision for this vulnerable age group. In addition to the current recommended minimum set of indicators, we suggest that additional indicators are collected periodically, for example through a network of sentinel PHCC or through household surveys [28]. The age and gender of those accessing services should be routinely recorded so that attendance by YP can be identified [21]. Additional training, support for clinic and school health team staff on data capture, which includes a focus on communicating why the data are being collected, and how the data will be used [29] may improve the accuracy and completeness of data. Timely feedback of data to clinic and school health team staff could inform planning and management of services for YP.

## Supplementary information

**Additional file 1: Table S1**. Provisional diagnoses/presenting condition among 10–24 yr olds by gender and age group (Pink register data, 2 PHC, 6 months in 2015/16).

**Additional file 2: Table S2.** Treatment among 10–24 yr olds by gender and age group (Pink register data, 2 PHC, 6 months in 2015/16)

**Additional file 3: Table S3.** Number of records reviewed in the registers according to school. Information on the conditions identified among those screened/vaccinated were also recorded: oral health [14], minor ailments [10], intestinal worms [9], eye health [6], suspected TB [3], underweight [3], overweight [1], hearing [1], and psychosocial support [1]. A total of 36 (20.8%) of learners on the school health register were referred to other services: social worker [2], cardiac problems [3], trauma/injuries [1], urinary problems [3], dental carries [5], poor eyesight [5], clinic check-up [8], and to higher level (no reason given) [9].

## Data Availability

The datasets generated and/or analysed during the current study are available in the Africa Health Research Institute repository 10.23664/AHRI.HSYP.Data.
