# Supplementary material for: Primary healthcare and school health service utilisation by adolescents and young adults in KwaZulu-Natal, South Africa
Source: BMC Health Serv Res. 2019 Nov 28;19:905. doi: 10.1186/s12913-019-4559-2 (PMC6883644; doi:10.1186/s12913-019-4559-2)
Supplement: Supplementary file 1 — Additional file 1: Table S1. Provisional diagnoses/presenting condition among 10–24 yr olds by gender and age group (Pink register data, 2 PHC, 6 months in 2015/16). [file 12913_2019_4559_MOESM1_ESM.docx]

**Additional file 1**

**Table S1.Provisional diagnoses/presenting condition among 10-24 yr olds by gender and age group (Pink register data, 2 PHC, 6 months in 2015/16)**

**Including abdominal pains/cramps, headache, backache, general body pains, painful legs, dizziness, fatigue, poor appetite, heart palpitations, nausea/vomiting, general body malaise, common cold/flu.*

*†Including testing, diagnosis, treatment, follow-up, treatment failure.*

*ǂ PAP, dysmenorrhoea, spotting, PV bleeding, pregnancy testing*
