# Supplementary material for: Primary healthcare and school health service utilisation by adolescents and young adults in KwaZulu-Natal, South Africa
Source: BMC Health Serv Res. 2019 Nov 28;19:905. doi: 10.1186/s12913-019-4559-2 (PMC6883644; doi:10.1186/s12913-019-4559-2)
Supplement: Supplementary file 2 — Additional file 2: Table S2. Treatment among 10–24 yr olds by gender and age group (Pink register data, 2 PHC, 6 months in 2015/16) [file 12913_2019_4559_MOESM2_ESM.docx]

**Additional file 2.**

**Table S2: Treatment among 10-24 yr olds by gender and age group (Pink register data, 2 PHC, 6 months in 2015/16)**
