# Supplementary material for: Primary healthcare and school health service utilisation by adolescents and young adults in KwaZulu-Natal, South Africa
Source: BMC Health Serv Res. 2019 Nov 28;19:905. doi: 10.1186/s12913-019-4559-2 (PMC6883644; doi:10.1186/s12913-019-4559-2)
Supplement: Supplementary file 3 — Additional file 3: Table S3. Number of records reviewed in the registers according to school. Information on the conditions identified among those screened/vaccinated were also recorded: oral health [14], minor ailments [10], intestinal worms [9], eye health [6], suspected TB [3], underweight [3], overweight [1], hearing [1], and psychosocial support [1]. A total of 36 (20.8%) of learners on the school health register were referred to other services: social worker [2], cardiac problems [3], trauma/injuries [1], urinary problems [3], dental carries [5], poor eyesight [5], clinic check-up [8], and to higher level (no reason given) [9]. [file 12913_2019_4559_MOESM3_ESM.docx]

**Additional file 3.**

**Table S3: Number of records reviewed in the registers according to school**

|  |  | **Number of records** | | | **Grade 8 screen** | | **Grade 10 screen** | | **12 year tetanus & diphtheria vaccine given** | |
| --- | --- | --- | --- | --- | --- | --- | --- | --- | --- | --- |
| **School number** | **Date of register** | **Females** | **Males** | **Total** | **F** | **M** | **F** | **M** | **F** | **M** |
| 1 | 11^th^/12^th^ Nov 15 | 21 (64%) | 12 (36%) | 33 (15.8%) | 0 | 0 | 0 | 0 | 21 | 12 |
| 2 | 20^th^ Nov 15 | 1 (100%) | 0 | 1 (0.5%) | 0 | - | 0 | - | 1 | - |
| 3 | 18^th^ & 22^nd^ Jan 16 | 81 (84%) | 16 (16%) | 97 (46.4%) | 50 | 9 | 22 | 7 | 41 | 7 |
| 4 | 27th Jan 16 | 0 | 6 (100%) | 6 (2.9%) | - | 0 | - | 0 | - | 6 |
| 5 | 5^th^, 7^th^, 8^th^ May 15 | 32 (73%) | 12 (27%) | 44 (21.1%) | 1 | 0 | 30 | 11 | 0 | 0 |
| 6 | 7^th^, 12^th^ & 13^th^ May 15 | 0 | 3 (100%) | 3 (1.4%) | - | 0 | - | 0 | - | 3 |
| 7 | 21^st^ & 24^th^ Jul 15 | 12 (48%) | 13 (52%) | 25 (12.0%) | 9 | 8 | 3 | 5 | 5 | 4 |
| TOTAL |  | 147 (70.3%) | 62 (29.7%) | 209 | 60 | 17 | 55 | 23 | 68 | 32 |

Information on the conditions identified among those screened/vaccinated were also recorded: oral health (14), minor ailments (10), intestinal worms (9), eye health (6), suspected TB (3), underweight (3), overweight (1), hearing (1), and psychosocial support (1). A total of 36 (20.8%) of learners on the school health register were referred to other services: social worker (2), cardiac problems (3), trauma/injuries (1), urinary problems (3), dental carries (5), poor eyesight (5), clinic check-up (8), and to higher level (no reason given) (9).
